# Integrity of the *Escherichia coli* O157:H7 Cell Wall and Membranes After Chlorine Dioxide Treatment

**DOI:** 10.3389/fmicb.2020.00888

**Published:** 2020-05-15

**Authors:** David F. Bridges, Alison Lacombe, Vivian C. H. Wu

**Affiliations:** Produce Safety and Microbiology Research Unit, Western Regional Research Center, Agricultural Research Service, United States Department of Agriculture, Albany, CA, United States

**Keywords:** chlorine dioxide, antimicrobial treatments, oxidizers, *Escherichia coli* O157:H7, bacterial membranes

## Abstract

Treatments of wastewater and fresh produce commonly employ chlorine as an antimicrobial. However, there are increasing levels of concerns regarding the safety and antimicrobial efficacy of chlorine treatments. Numerous studies have reported the antimicrobial properties of chlorine dioxide (ClO_2_) treatment in a variety of applications but information regarding how ClO_2_ affects bacteria is limited. In the present study, a mixed-method approach utilizing both quantitative and qualitative methodologies was used to observe *Escherichia coli* O157:H7 membrane damage after exposure to ClO_2_ (2.5, 5, or 10 mg/L) for 5, 10, or 15 min. For comparison, controls of 0.1% peptone, 70% isopropanol, and 10 mg/L NaOCl were applied for 15 min. After treatment, cells were enumerated on selective media overlaid with non-selective media and simultaneously analyzed for damage using the following fluorescent probes (1) Bis-(1,3-Dibutylbarbituric Acid) trimethine oxonol (DiBAC4(3)) for membrane polarization, (2) SYTO 9/propidium iodide (LIVE/DEAD) for membrane permeability, (3) 2-(N-(7-Nitrobenz-2-oxa-1,3-diazol-4-yl)Amino)-2-Deoxyglucose (2-NBDG) for active glucose uptake, and (4) lipid peroxidation through accumulation of malondialdehyde (MDA). Bacterial log reductions after ClO_2_ treatment ranged from 0.2 to 5.5 and changes in relative fluorescence units after membrane permeability and glucose uptake assays were not consistent with viability, indicating membrane permeability and metabolism were not substantially altered. Depolarization was observed after NaOCl treatment, however, the polarity of cells treated with ClO_2_ were like those treated with water (*P* < 0.05). Accumulation of MDA was detected only after 10 mg/L ClO_2_ treatments, indicating that membrane peroxidation occurred at higher concentrations. Transmission electron microscopy imaging revealed that separation of the cell wall from the cytosol occurred after the 10 mg/L ClO_2_ treatment, but the cell wall itself appeared to be unbroken. These data suggest that ClO_2_ damage to *E. coli* O157:H7 is not primarily located at the cell wall and harms cells significantly different than NaOCl at comparable concentrations.

## Introduction

Chlorine in the form of sodium hypochlorite (NaOCl) or calcium hypochlorite [Ca(OCl_2_)], is the most commonly used antimicrobial for produce washes and water sanitation. However, free chlorine in wash water is consumed rapidly by the presence of any organic matter (e.g., soil or plant tissues) which makes maintaining a constant concentration during sanitation processes a challenge (Gil et al., 2009). Additionally, chlorinated washes lack efficacy outside a narrow pH range and can potentially form carcinogenic substances, such as trichloramines, as byproducts of treatment (Richardson et al., 1998; Shen et al., 2016). The use of chlorine dioxide (ClO_2_) washes as an alternative to conventional chlorinated washes has increased in popularity over the past 20 years due to their high oxidative capacity, stability over a wide pH range, antimicrobial efficacy, and limited toxic byproduct formation (Gómez-López et al., 2009; Wu, 2016; Bridges and Wu, 2018; Praeger et al., 2018; Tadepalli et al., 2018; Sun et al., 2019). Currently, ClO_2_ is used for applications such as disinfection of drinking water and wastewater, antimicrobial agent in water used in poultry processing and fruits and vegetables that are not raw agricultural commodities, sterilization of medical surfaces, equipment, and waste, and bleaching in pulp and paper industries (Gómez-López et al., 2009; Praeger et al., 2018).

As an antimicrobial agent, ClO_2_ washes have been previously demonstrated to effectively reduce levels of pathogenic bacteria including, but not limited to, Shiga toxin-producing *Escherichia coli*, *Salmonella enterica, Listeria monocytogenes*, *Pseudomonas aeruginosa, Staphylococcus aureus*, and *Yersinia enterocolitica* on a variety of produce (Gómez-López et al., 2009; Wu, 2016; Praeger et al., 2018; Tadepalli et al., 2018; Bridges et al., 2019). Nevertheless, information regarding how ClO_2_ affects bacterial cells is limited. At the cell surface level, ClO_2_ is believed to oxidize sensitive sulfhydryl groups on cell-surface proteins which results in membrane damage and increased outer membrane permeability (Berg et al., 1986; Praeger et al., 2018). Previous studies that treated *E. coli*, *P. aeruginosa*, and *S. aureus* with ClO_2_ (≤5 mg/L) achieved maximum log reductions of >5 log CFU/mL and found that after treatment there was an increase in bacterial membrane permeability. However, additional examination of transmission electron microscopy (TEM) micrographs further showed that there was no noticeable morphological or cell wall damage (Ofori et al., 2018, 2017).

Alternatively, ClO_2_ is membrane permeable and has previously been shown to react with amino acids (Sharma and Sohn, 2012) indicating that bacterial lethality could be due to oxidation of internal proteins and nucleic acids or disruption of protein synthesis. Bacterial membranes and cell walls would not be directly damaged by ClO_2_ if this were the case. It is also possible that cell death could be caused by accumulation of different damage from widespread oxidation rather than damage to a single region or area. Furthermore, it is difficult to determine if observed damages (e.g., altered membrane permeability) to bacteria after treatment were truly caused by ClO_2_ or if they were due to the natural decay of large amounts of dead bacteria.

While the capacity of ClO_2_ to serve as an antimicrobial in a variety of applications has been established, the types of damages that result in bacterial lethality are unclear. Therefore, the objective of this study is to observe the damage at the membrane interface of *E. coli* O157:H7 after treatment with ClO_2_ using a both quantitative and qualitative methods. Comparing physiological metrics using over a range of treatment efficacies (e.g., 0–5 log reductions) could clarify if membrane damage is the primary cause of bacterial cell death and improve understanding of the antibacterial properties of ClO_2_.

## Materials and Methods

### Preparation of Bacterial Culture

A 10 mL tube of tryptic soy broth (TSB) was inoculated and incubated overnight at 37°C using a frozen stock culture of *E. coli* O157:H7 (ATCC^®^ 35150) maintained at −80°C. Only one strain of *E. coli* was selected to reduce variance in collected data due to differences between strains. This overnight culture was used to inoculate slants of tryptic soy agar (TSA) which were incubated overnight at 37°C and kept as working cultures throughout the study at 4°C. A day prior to experimentation, a loopful of *E. coli* O157:H7 culture maintained on TSA was used to inoculate 10 mL tubes of TSB which were then incubated for 16–18 h at 37°C. After incubation, the cultures were centrifuged for 10 min at 10,000 × *g* and the resultant pellet was washed twice with 0.1% peptone water and resuspended in 9 mL of 0.1% peptone water.

### Generation of Aqueous ClO_2_

Chlorine dioxide solutions were generated using a two-component dry media system provided by ICA TriNova, LLC (Forest Park, GA, United States). Following manufacturer protocol, sodium chlorite (NaClO_2_) and a proprietary activating acid were combined in a sachet and place into 7.57 L (2 gal) of distilled water and stored protected from light at room temperature (21°C) for 5 days. After, the sachet was removed and the ClO_2_ stock solution was stored in the dark at 4°C for future use. The concentrations of this stock solutions and the ClO_2_ solutions used for treatment in this study were measured before each experiment by the DPD (N,N-diethyl-r-phenylenediamine) method using a Hatch DR 900 colorimeter as described previously (Wu and Kim, 2007; Tadepalli et al., 2018).

### Treatment of *E. coli* O157:H7 With ClO_2_ and Measurement of Viability

Chlorine dioxide treatment times and concentrations were selected based on commonly utilized conditions in published literature (Wu and Kim, 2007; Tadepalli et al., 2018). Before treatment, the ClO_2_ stock solution was diluted with deionized (DI) water to make working solutions of 25, 50, and 100 mg/L ClO_2_. One milliliter of these working ClO_2_ solutions was then added to 9 mL of prepared *E. coli* O157:H7 cultures to make treatments with final concentrations of 2.5, 5, and 10 mg/L. Treatments were conducted for 5, 10, or 15 min and the cultures were vortexed every 5 min. To end the treatments, 1 mL of 1% sodium thiosulfate was added to the tubes (making a 0.09% solution) to inactivate remaining ClO_2_. Deionized water or isopropanol (70% final concentration) were included as negative and positive treatment controls, respectfully and NaOCl (10 mg/L final concentration) was included for comparison to ClO_2_ at equivalent concentrations. The control treatments were conducted for 15 min for comparison to ClO_2_ after the longest exposure times. After treatment, the solutions were serially diluted in 0.1% peptone water and plated on MacConkey’s sorbitol agar supplemented with 0.05 mg/L cefixime and 2.5 mg/L potassium tellurite (CT-SMAC) and overlaid with TSA (Thin Agar Layer method) to help recover sub-lethally injured bacteria as described previously (Wu, 2008).

### Preparation of Bacteria for Fluorescent Assays

After treatment and inactivation with sodium thiosulfate, solutions were centrifuged at 10,000 × *g* for 10 min. The supernatant was removed and the pellet was re-suspended in 0.1% peptone water. This washing step was repeated and the final pellet was suspended in 1 mL of 0.1% peptone water.

### Measurement of Metabolic Activity With 2-NBDG

Intracellular uptake of 2-(N-(7-Nitrobenz-2-oxa-1,3-diazol-4-yl)Amino)-2-Deoxyglucose (2-NBDG), a fluorescent analog of deoxy-glucose, has been previously utilized as in indicator of metabolic activity via glucose uptake (Yoshioka et al., 1996; Cossu et al., 2017). Following the methodology used by Cossu et al. (2017), 5 μL of 1 mg/mL 2-NBDG in dimethyl sulfoxide (DMSO) was added to 995 μL of treated sample, making a final concentration of 5 μg/mL, and was incubated in the dark for 20 min at 37°C. After incubation, 200 μL of the stained cultures were added to wells in a 96-well plate and read in a fluorescence plate-reader at 465/540 nm.

### Measurement of Bacterial Membrane Permeability Using LIVE/DEAD Staining

Following a similar procedure similar to that used by Lacombe et al. (2013a, b), the cell membrane permeability of *E. coli* O157:H7 after treatment was assessed using the LIVE/DEAD BacLight Bacterial Viability Kit (Invitrogen, Grand Island, NY). The LIVE/DEAD method uses two different nucleic acid stains, SYTO 9 and propidium iodide (PI) to help differentiate between live and dead cells. Working solutions of 3 mM SYTO 9 and PI in DMSO were diluted to 0.3 mM in DI water and 50 μL of each stain were added to wells in a 96-well plate. One hundred microliters of treated bacteria were added to staining solutions in 96-well plates and incubated in the dark at RT for 15 min. After incubation, plates were read at 485/498 nm and 535/617 nm for SYTO 9 and PI, respectfully.

### Observing Changes in Bacterial Membrane Polarization Using DiBAC_4_(3)

Bis-(1,3-Dibutylbarbituric Acid) Trimethine Oxonol (DiBAC_4_(3)) is an anionic potential-response membrane probe that has been previously used to monitor changes in membrane polarization. Following the protocol utilized by Lacombe et al. (2013a), a 5 mM DiBAC_4_(3) solution in DMSO was diluted to 0.5 mM in DI water and 100 μL of stain was added to 100 μL of treated samples in a 96-well plate and incubated at RT in the dark for 45 min. After 45 min, fluorescent intensity was measured at 490/516 nm in a plate reader.

### Detection of Bacterial Lipid Peroxidation Through MDA Detection

Lipid peroxidation was measured using a Lipid Peroxidation (MDA) Kit (Abcam, Cambridge, MA, United States) following the manufacturer’s protocol. In short, 300 μL of lysis buffer and 3 μL of butylhydroxytoluene were added to 1 mL of recovered bacterial solution, vortexed for 1 min, and then centrifuged at 13,000 × *g* for 10 min. After centrifugation, 200 μL of supernatant was removed and added to 600 μL of previously prepared thiobarbituric acid (TBA) reagent and incubated for 60 min at 95°C. After incubation, the solutions were cooled for 10 min in an ice bath and the relative fluorescence units (RFU) at 532/553 nm was taken using a plate reader. The concentration of the MDA-TBA adduct (nmol) was then determined by comparing the measured RFU against a previously generated MDA standard curve.

### Transmission Electron Microscopy Preparation and Imaging

*Escherichia coli* after 0.1% peptone water, 70% isopropanol, 10 mg/L NaOCl, and 2.5, 5, or 10 mg/L ClO_2_ treatment for 15 min were selected for TEM examination. The 15 min time point was selected to represent the state of the cells after the most severe treatment stresses. Bacteria recovered after treatment were preserved overnight in standard fixative (2.5% glutaraldehyde, 2% formaldehyde, 2.5 mM CaCl_2_, in 0.1 M sodium cacodylate; pH 6.9) and then washed three times with 0.1 M sodium cacodylate for 20 min and stained with 1% osmium tetroxide (OsO_4_) for 2 h. After staining, the samples were washed three times with 0.1 sodium cacodylate for 20 min and dehydrated with a degrading acetone series (30, 50, 75, 95, 100, 100, 100%; 30 min each). The dehydrated samples were imbedded in Spurrs’s resin and hardened at 40°C for 1 day followed by 1 week at 60°C. Ultra-thin sections (50–100 nm) of the prepared resin blocks were made using a microtome, transferred to 200-mesh copper grids, stained with 2% uranyl acetate for 30 min and 0.5% lead citrate for 1 min, and viewed using a JOEL 1200 EX transmission electron microscope at an accelerating potential of 80 kV (Bozzola, 2014).

### Data Analysis

All experiments were performed in biological triplicate (*n* = 3) and statistical analysis was performed using JMP (ver. 12) or Sigmaplot (ver. 14) software with α = 0.05. Log reductions of bacteria were determined by subtracting the populations of bacteria recovered after treatment from untreated controls. One-way ANOVAs coupled with Tukey’s HSD *post hoc* tests were used to determine significant differences among the treatment conditions for each assay. Spearman’s rank correlation coefficients between the results of each assay after ClO_2_ treatment were calculated using the linear regressions found for each concentration over the time points.

## Results and Discussion

### Bacterial Viability After Treatment

**FIGURE 1:**
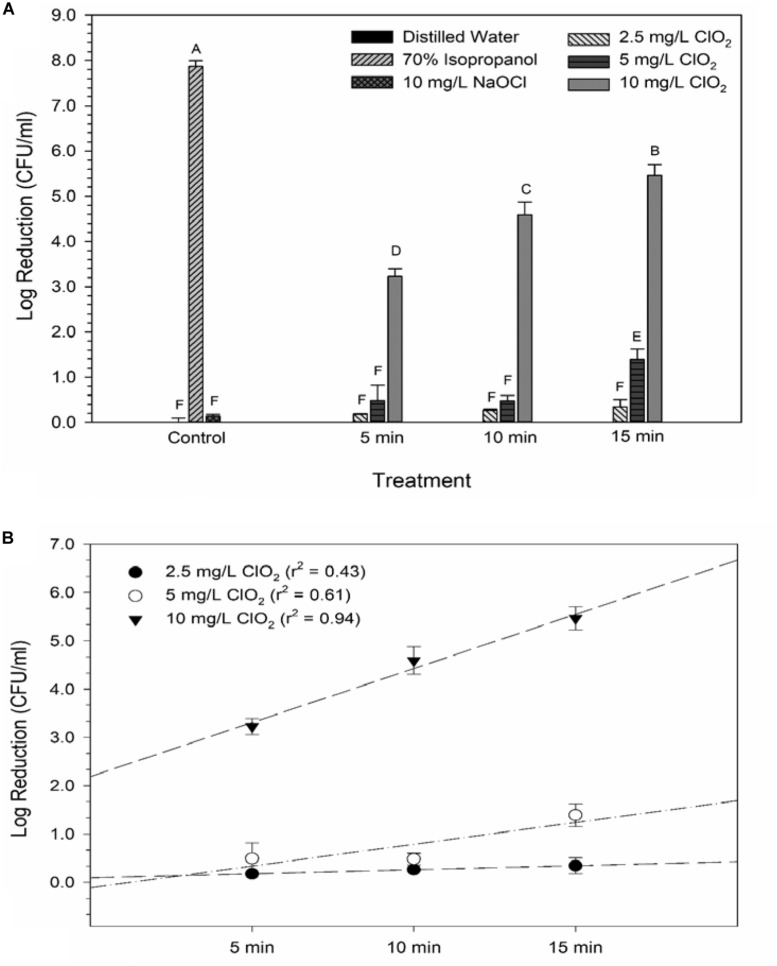
Log reduction of *E. coli* O157 after 2.5, 5, or 10 mg/L ClO_2_ treatment for 5, 10, or 15 min compared to control treatments **(A)** and linear regressions of bacterial reduction over time after ClO_2_ treatment **(B)**. Data are presented as means ± standard deviations (*n* = 3) and significant differences (*P* ≤ 0.05) in bacteria reductions observed after each treatment are represented by different letters (e.g., A–F).

The average reductions of *E. coli* O157:H7 after ClO_2_ or control treatments are presented in Figure 1. Maximum log reductions of 0.3, 1.4, and 5.5 were achieved after 15 min exposure to 2.5, 5.0, or 10 mg/L ClO_2_, respectively. Treatments with 2.5 mg/L ClO_2_ for all three time points and 5.0 mg/L treatment for 5 and 10 min resulted in reductions similar to DI water and 10 mg/L NaOCl treatments. The ClO_2_ or NaOCl could have reacted with the peptone water which would subsequently lower antibacterial efficacy. However, the 10 mg/L ClO_2_ treatments had significantly (*P* < 0.05) different log reductions compared to the other two concentrations. There was a positive relationship (*r*^2^ = 0.94) between bacterial reduction and exposure time, indicating that reduction of *E. coli* O157:H7 after treatment with 10 mg/L ClO_2_ solutions could be a function of exposure time.

### Changes in Metabolic Activity Measured by 2-NBDG Degradation

**FIGURE 2:**
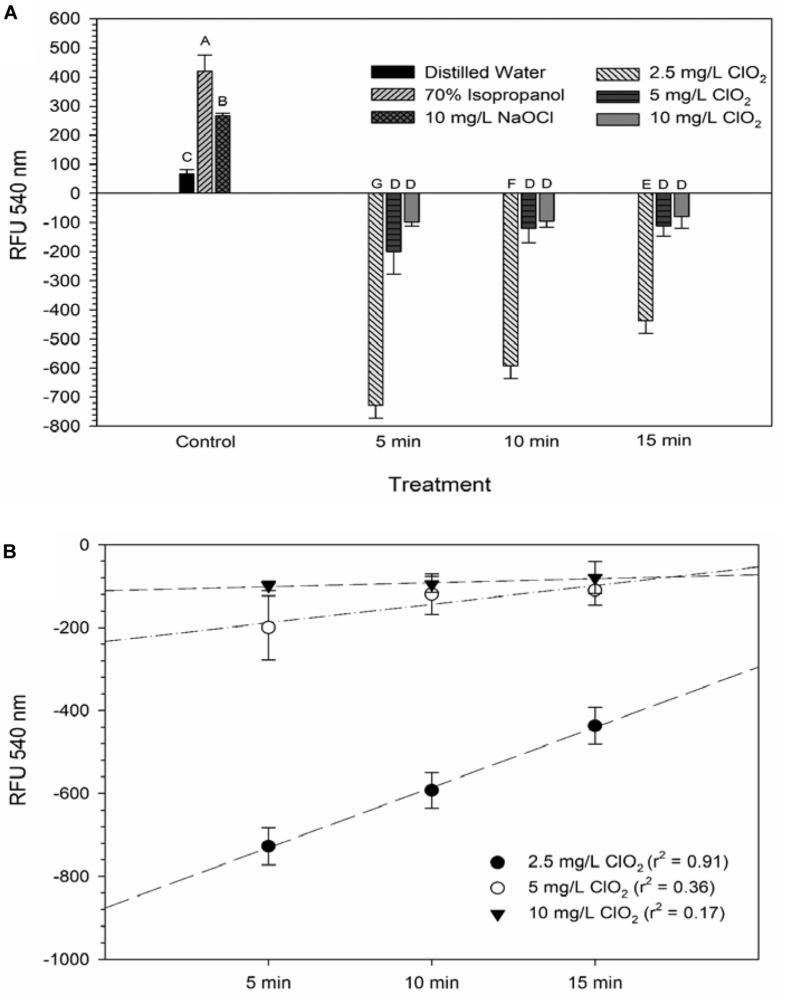
2-NBDG signal after 2.5, 5, or 10 mg/L ClO_2_ treatment for 5, 10, or 15 min compared to control treatments **(A)** and linear regressions of 2-NBDG signal over time after ClO_2_ treatment **(B)**. Data are presented as means ± standard deviations and significant differences (*P* ≤ 0.05) in bacteria reductions observed after each treatment are represented by different letters (e.g., A–G).

Glucose is a simple sugar that is an important source for all living organisms and metrics of glucose uptake by bacteria can provide information pertaining to cell viability and metabolism. In principal, healthy cells will take up the stain and normal metabolic processes cleave the fluorescent unit and leave a non-fluorescent product, thus causing a decrease in fluorescent signal. If antimicrobial treatment causes damage to the proteins or channels involved in glucose uptake or metabolism (e.g., the phosphotransferase system), a stronger fluorescent signal could be expected. All treatments with ClO_2_ resulted in negative RFUs, indicating that there was 2-NBDG degradation which could be indicative of increased metabolic activity (Figure 2). Furthermore, this phenomenon was not observed after any of the control treatments. The 5 and 10 mg/L treatments had similar (*P* ≥ 0.05) decreases in 2-NBDG across all treatment times, indicating that exposure time did not have a strong effect on 2-NBDG metabolism for these concentrations. However, the 2.5 mg/L treatment had significant (*P* < 0.05) increases in RFU at each time point and a positive linear correlation with increase in exposure time (*r*^2^ = 0.91), indicating a decrease in glucose metabolism over time. Previously, Cossu et al. (2017) demonstrated that 2-NBDG uptake by *E. coli* O157 was correlated with viability after treatment with NaOCl and H_2_O_2_. An increased flux of glucose can increase production of NADPH through the pentose phosphate pathway which allows for a higher capacity of NADPH-mediated reductive detoxifying reactions (Gray et al., 2013). However, there was not a significant correlation between glucose uptake and viability found after treatment with 2.5, 5, or 10 mg/L ClO_2_ (Table 1). If increased glucose uptake improves survivability during oxidative stress, then a positive correlation between recovery and 2-NBDG would be expected. Previously, ClO_2_ has been shown to rapidly react with NADH (Bakhmutova-Albert et al., 2008) and if ClO_2_ in this study was degrading the pool of NADH, then there would predictably be a decrease in ATP generation, subsequently hindering most cellular processes.

**TABLE 1:** Spearman rank order correlation coefficients from comparison of viability, LIVE/DEAD, 2-NBDG, and DiBAC_4_(3) data after exposure to 2.5, 5, or 10 mg/L ClO_2_ across the treatment times.

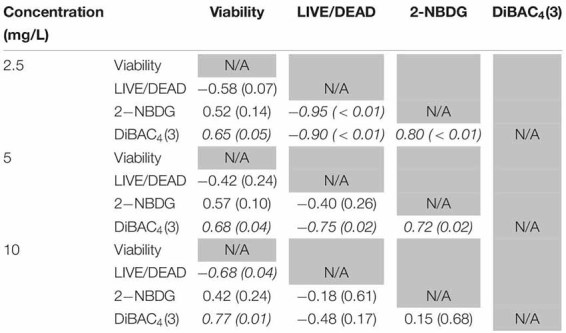	

*Data are presented as Spearman Correlation Coefficient (SCC) followed by the *P*-value [SCC(*P*-value)] and significant comparisons (*P* < 0.05) are indicated by italicized text. The gray boxes represent crossings that are represented elsewhere on the table.*

### Changes in Membrane Permeability Measured by LIVE/DEAD Assay

**FIGURE 3:**
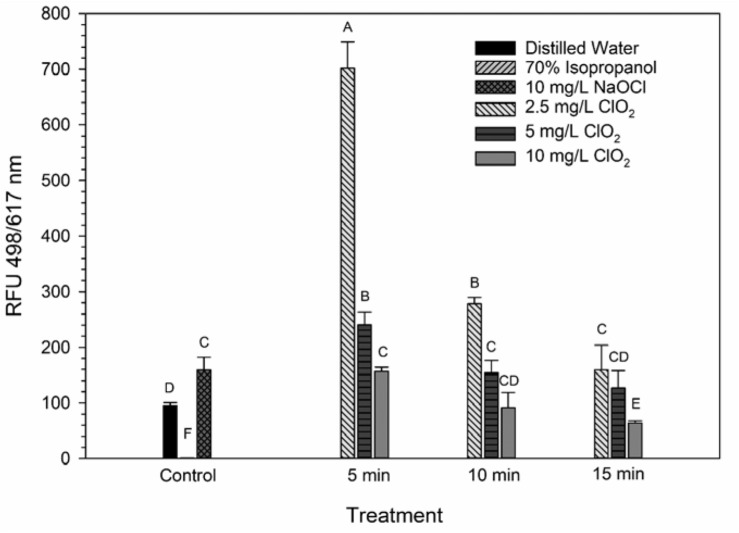
SYTO 9: PI ratio after 2.5, 5, or 10 mg/L ClO_2_ treatment for 5, 10, or 15 min compared to control or ClO_2_ treatment. Data are presented as means ± standard deviations (*n* = 3) and significant differences (*P* ≤ 0.05) in bacteria reductions observed after each treatment are represented by different letters (e.g., A–F).

The LIVE/DEAD method uses two different nucleic acid stains, SYTO 9 and propidium iodide (PI), to differentiate between live and dead cells. SYTO 9 is membrane permeable and will typically stain all cells within a population while PI can only enter cells that have damaged membranes (Rosenberg et al., 2019). The ratio of SYTO 9:PI signal consistently decreased over exposure times and most of the treatment conditions did not result in signal ratios significantly different from the distilled water or NaOCl treatments (Figure 3). The changes in LIVE/DEAD RFU over the treatment times had a significant inverse correlation (*P* = 0.04) with viability during the 10 mg/L ClO_2_, but not 2.5 or 5 mg/L treatments (Table 1). These results could suggest that sub-lethal changes in membrane permeability might have been occurring, which would influence cellular metabolism and energy transduction. Additionally, the notable increase of SYTO 9 signal after the 2.5 mg/L treatment (Supplementary Figure 1) is indicative of an increase in RNA levels.

Ofori et al. (2017, 2018) demonstrated that *E. coli* and *P. aeruginosa* treated with 2.5 and 5 mg/L ClO_2_ had significant increases of 1-N-phenylnaphthylamine (NPN) uptake, a typically membrane impermeable probe, indicating that there was a clear change in cell membrane permeability. Furthermore, when viewed under a TEM, the cells were not lysed and did not have apparent morphological damage, implying that improved permeability of the bacterial membrane could be a significant cause of cell death. Conversely, in the present study there was not a noticeably similar increase in PI signal after ClO_2_ treatment (Supplementary Figure 2). These data imply that any alternations in membrane permeability that occurred from ClO_2_ treatment were not substantial enough to allow for PI to enter the cells. Because NPN binds to hydrophobic regions of cell membranes and PI binds to nucleic acids, the increased binding of NPN observed by Ofori et al. (2017, 2018) could represent increased permeability limited to the gram-negative outer membrane. If there was an increase in the permeability of both the outer and inner cell membranes, then an increase in PI binding would be expected. It is also important to note that in the present study the bacterial cells were in 0.1% peptone water during treatment while Ofori et al. (2017, 2018) performed their treatments in oxidant demand free buffered water. The differences between these two mediums could have also influenced the effect that ClO_2_ treatment has on membrane permeability.

### Changes in Membrane Polarity Measured by DiBAC_4_(3) Staining

**FIGURE 4:**
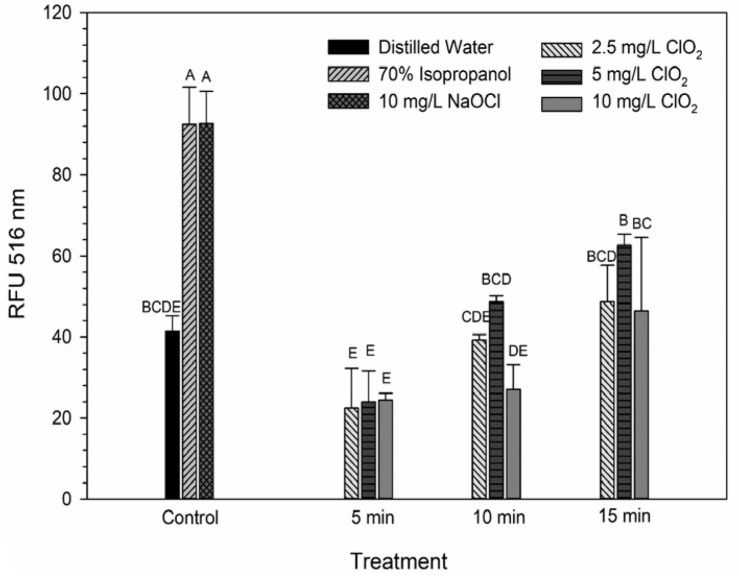
DiBAC_4_(3) uptake after 2.5, 5, or 10 mg/L ClO_2_ treatment for 5, 10, or 15 min compared to control or ClO_2_ treatment. Data are presented as means ± standard deviations (*n* = 3) and significant differences (*P* ≤ 0.05) in bacteria reductions observed after each treatment are represented by different letters (e.g., A–E).

DiBAC_4_(3) is an anionic potential-response membrane probe that is typically excluded by healthy cells. If damage occurs that alters membrane polarization, DiBAC_4_(3) can enter cells and bind to intracellular proteins and membrane where it exhibits increased fluorescence. The bacteria treated with isopropanol or NaOCl had significantly (*P* < 0.05) different higher levels of DiBAC_4_(3) binding than any of the ClO_2_ treatments or the DI water control (Figure 4), but there were no significant differences after the ClO_2_ treatments compared to the DI water treated cells. Correlations between changes in DiBAC_4_(3) signal and viability, LIVE/DEAD, and 2-NBDG data after the 2.5 and 5 mg/L ClO_2_ treatments were observed (Table 1). Furthermore, at the 10 mg/L concentration, only viability was correlated with DiBAC_4_(3) which could indicate that membrane polarization is still maintained by cells up until death. In contrast, both the NaOCl and isopropanol treatments had similar DiBAC_4_(3) levels, yet, the isopropanol killed a substantial number of cells while the NaOCl caused a negligible level of reduction. The membrane polarization needed for oxidative phosphorylation in gram negative bacteria is caused by the active transport of protons from the cytosol to the periplasm across the inner membrane. At equivalent concentrations (10 mg/L), NaOCl caused a significant disruption of this polarization while ClO_2_ did not. There is strong evidence that NaOCl treatment damages bacterial membranes, which would interfere with maintaining proton gradients (Virto et al., 2005; Gray et al., 2013; Cossu et al., 2017). In solutions NaOCl breaks down into membrane permeable hypochlorous acid (HOCl) and membrane impermeable hypochlorite (OCl^–^). As such, the critical factor determining antimicrobial effectiveness of NaOCl treatment is the concentration of HOCl because it is capable of permeating through bacterial membranes and causing intracellular oxidative damage (Fukuzaki, 2006). Chlorine dioxide is also membrane permeable, but at equal concentrations did not cause similar levels of membrane depolarization as NaOCl. Therefore, it appears that the antibacterial mechanism of ClO_2_ is different NaOCl. This could be due to ClO_2_ having a much lower oxidative strength than HOCl while simultaneously having a much higher oxidative capacity (Wu, 2016; Bridges and Wu, 2018).

### Presence of MDA After Treatment and TEM Imaging

**FIGURE 5:**
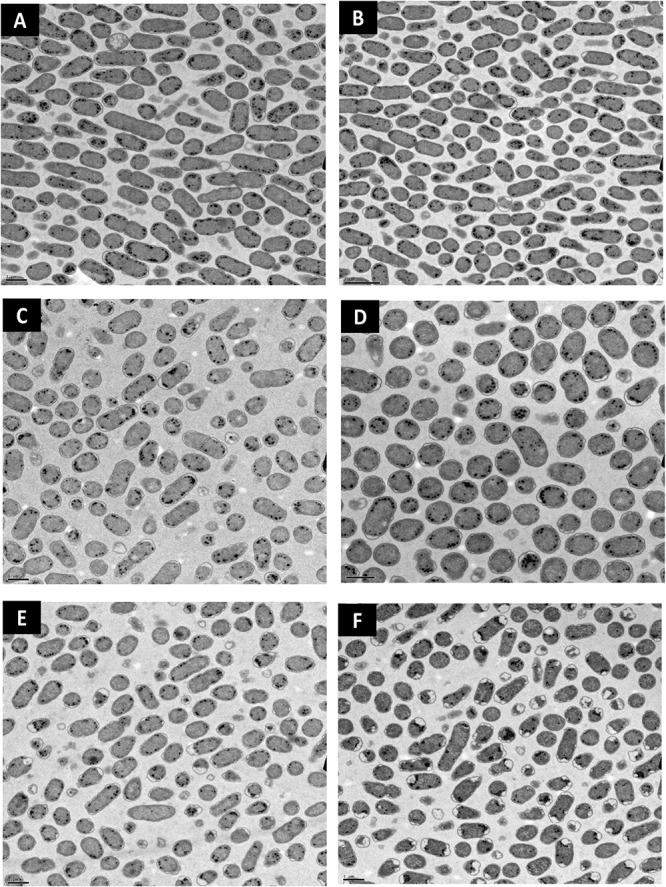
Transmission electron micrographs of *E. coli* O157 after treatment with distilled water **(A)**, 70% isopropanol **(B)**, 10 mg/L NaOCl **(C)**, 2.5 mg/L ClO_2_ **(D)**, 5 mg/L ClO_2_ **(E)**, or 10 mg/L ClO_2_ **(F)** for 15 min.

**FIGURE 6:**
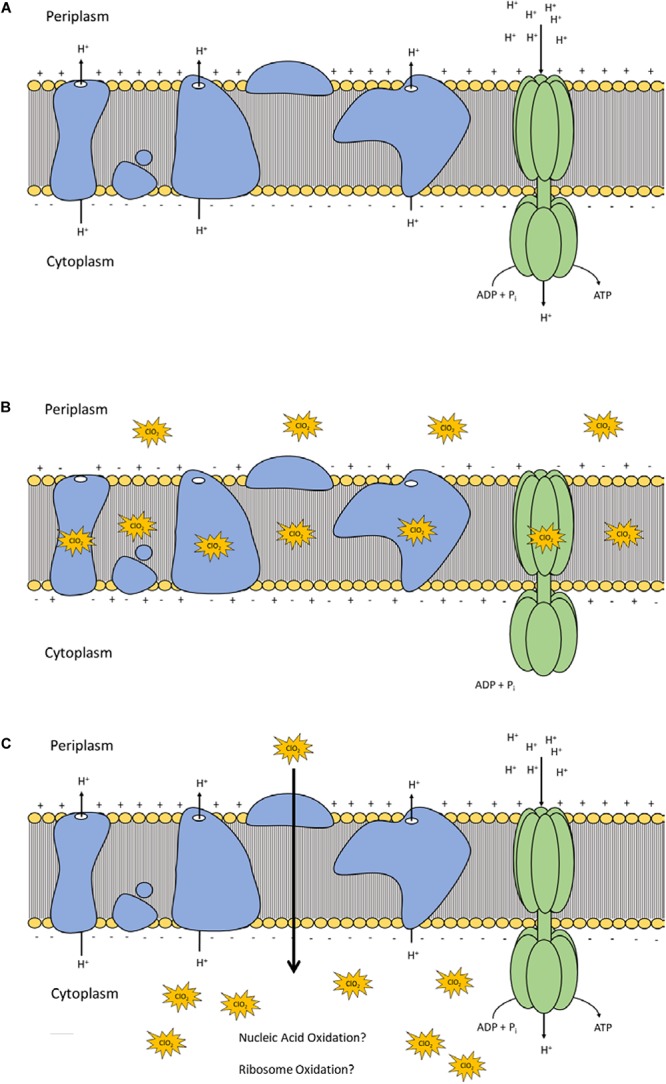
Polarization and integrity schematic of the *E. coli* cytoplasmic membrane during normal conditions **(A)** and after ClO_2_ treatment if damage is localized at membrane proteins and lipids **(B)** or if ClO_2_ damage is localized intracellularly **(C)**.

Lipid peroxidation is a commonly used measure of oxidative stress. In principal, oxidation of unsaturated fatty acids in the lipid bilayer would produce MDA which can be measured spectrophotometrically (Lefevre et al., 1998). If ClO_2_ was causing oxidative damage to the lipid bilayer, then accumulation of MDA should start occurring in sublethal conditions. Yet, only the 10 mg/L ClO_2_ treatment resulted in measurable levels of MDA in *E. coli* O157 cells (Table 2). Additionally, the maintenance of membrane polarization after treatment with 10 mg/L ClO_2_ indicates that lipid oxidation did not significantly disrupt oxidative phosphorylation and likely occurred at the outer membrane or after cellular death. Examination of the cells using TEM revealed that after treatment with 10 mg/LClO_2_ the bacterial cell wall started to separate from the cytosol (Figure 5). This observation, combined with the results from the DiBAC_4_(3) assay, indicate that the primary damage to *E. coli* O157:H7 after treatment occurred intracellularly and left the cell wall and membrane primarily intact. Ofori et al. (2017) also previously observed little morphological changes in *E. coli* after treatment with ClO_2_ (2.0 mg/L). However, they also measured an increase of 240 nm absorbing materials after treatment which indicates a disruption or increase in permeability of the cytoplasmic membranes. If a significant amount of damage was occurring at the cell wall and membrane level, then there should be an alteration of membrane polarity that was not observed in this study. These findings indicate that the primary lethal damage from ClO_2_ is localized intracellularly and any changes in membrane permeability were sublethal (Figure 6). Furthermore, the increased intake of glucose and exclusion of PI suggest that the functions of the cell wall and membranes are maintained during ClO_2_ exposure. Given that these results are different than what Ofori et al. (2017, 2018) previously observed with *E. coli* and *P. aeruginosa*, it could be possible that ClO_2_-induced damage could be localized differently depending on the species of bacteria or even the strain.

**TABLE 2:** Detection of MDA after treatment with controls or ClO_2_.

	**Treatment**	**Detected MDA (nmol)**
Controls	Distilled Water	<0.1
	70% Isopropanol	<0.1
	10 mg/L NaOCl	<0.1
Chlorine Dioxide	2.5 mg/L (5 min)	<0.1
	2.5 mg/L 10 min)	<0.1
	2.5 mg/L (15 min)	<0.1
	5 mg/L (5 min)	<0.1
	5 mg/L (10 min)	<0.1
	5 mg/L (15 min)	<0.1
	10 mg/L (5 min)	3.25 ± 0.02
	10 mg/L (10 min)	3.28 ± 0.02
	10 mg/L (15 min)	3.30 ± 0.02

*Data are presented as means ± standard deviation.*

## Conclusion

In this study the damage of the cell wall and membranes of *E. coli* O157:H7 were observed after treatment with ClO_2_ using a mixed-method approach combining both quantitative and qualitative methods. After treatments that resulted in >5 log reductions, the polarity of the cell membrane was measured to be maintained and there were not atypical changes in glucose uptake or membrane permeability. While separation of the cell wall from the cytosol was observed after treatment, the results from multiple assays imply that the integrity of the cell wall remained intact. Therefore, ClO_2_-damage of *E. coli* O157:H7 appears to be localized intracellularly which helps clarify discrepancies in the literature and increase the understanding of the “mode-of-action” behind the notable antibacterial efficacy of ClO_2_ treatment.

## Data Availability Statement

All datasets generated for this study are included in the article/Supplementary Material.

## Author Contributions

DB preformed the experiments and data analysis. VW was responsible for funding acquisition, project supervision, and project administration. DB, AL, and VW contributed to manuscript writing.

## Conflict of Interest

The authors declare that the research was conducted in the absence of any commercial or financial relationships that could be construed as a potential conflict of interest.
