# Supplementary material for: Integrity of the Escherichia coli O157:H7 Cell Wall and Membranes After Chlorine Dioxide Treatment
Source: Front Microbiol. 2020 May 15;11:888. doi: 10.3389/fmicb.2020.00888 (PMC7243733; doi:10.3389/fmicb.2020.00888)
Supplement: Supplementary file 1 [file Data_Sheet_1.docx]

**Supplemental Figure 1.** SYTO 9 signal after 2.5, 5, or 10 mg/L ClO_2_ treatment for 5, 10, or 15 min compared to control treatments.





**Supplemental Figure 2.** Propidium iodide signal after 2.5, 5, or 10 mg/L ClO_2_ treatment for 5, 10, or 15 min compared to control treatments.
